# A comparative study on tobacco prevalence and secondhand smoke exposure before and after the lockdown in Rizhao, China: analysis of 2022 and 2024 data

**DOI:** 10.3389/fpubh.2025.1588781

**Published:** 2025-06-26

**Authors:** Zhao-Hui Liang, Gui-Zhi Han, Miao-Miao Liu

**Affiliations:** School of Public Health, Jining Medical University, Jining, Shandong, China

**Keywords:** tobacco epidemic, smoking, passive smoking, second-hand smoke, awareness, COVID-19

## Abstract

**Objective:**

This study aims to compare the trends of tobacco prevalence and secondhand smoke exposure in Rizhao, China, before and after the lockdown, and to analyze the changes in residents’ awareness of smoking-related diseases.

**Method:**

Two cross-sectional surveys on tobacco prevalence and secondhand smoke exposure were conducted in Rizhao, China, in 2022 and 2024. The chi-square test was used to determine whether there were significant differences.

**Result:**

A total of 1872 valid questionnaires were collected from the two surveys. The results showed that the current smoking rate declined significantly from 32.53 to 25.84% (*χ^2^* = 10.08, *p* = 0.002), with daily smoking rate also slightly decreased from 20.44 to 19.33% (*χ^2^* = 0.36, *p* = 0.551). Paradoxically, the passive smoking rate among nonsmokers surged from 32.08 to 48.39% (*χ^2^* = 35.53, *p* < 0.01), while residents’ awareness of smoking-related diseases declined.

**Conclusion:**

Our study reveals a paradoxical situation in Rizhao: declining active smoking contrasts sharply with escalating secondhand smoke exposure, along with decreasing public awareness of smoking-related diseases. These trends suggest that conventional tobacco control measures have been effective in curbing active smoking, while they are insufficient to address the new behavioral patterns emerging from the prolonged indoor confinement and changing work-life arrangements during the pandemic. Effective tobacco control campaigns and targeted interventions for key populations are urgently needed.

## 1 Introduction

In 2023, the World Health Organization (WHO) released the latest report on the Global Tobacco Epidemic, highlighting the devastating impact of tobacco use on global public health. According to the report, tobacco use is responsible for more than 8.7 million deaths annually worldwide, making it one of the most significant public health challenges endangering human health on a global scale (1). China has the largest smoking population in the world, with approximately 308 million smokers, and over one million Chinese citizens succumbing to tobacco-related diseases each year (2, 3). In the meanwhile, research has consistently demonstrated that exposure to secondhand smoke significantly increases the risk of mortality from heart disease, stroke, respiratory diseases, type 2 diabetes, and various forms of cancer. It is estimated that around 1.3 million people worldwide die annually due to passive smoking (4, 5).

The recent COVID-19 pandemic has exerted a profound impact on residents’ behavior patterns and cognitive concepts. An online survey study on Chinese smokers revealed that during the COVID-19 pandemic, 7.7% of smokers reported an increase in cigarette consumption, while 19.2% of respondents reported a decrease in smoking (6). However, a cross-sectional survey in Israel showed that during the pandemic, although many respondents believed that the use of nicotine products (especially combustible cigarettes and e-cigarettes) was associated with an increased risk of COVID-19 disease severity, the majority of users did not change their usage habits of tobacco/nicotine products (7). While extant studies have revealed the residents’ tobacco prevalence during the COVID-19 pandemic, the absence of comparative analysis between the pandemic and post-pandemic periods prevents a comprehensive understanding of the long-term impact of the pandemic on residents’ tobacco use behaviors. Meanwhile, the influence of the COVID-19 pandemic on the residents’ awareness of smoking-related diseases remains unclear.

This study aims to compare the tobacco epidemic and secondhand smoke exposure among residents in Rizhao, China, before and after the lockdown. Additionally, we will analyze the changes in residents’ awareness of smoking-related diseases and passive smoking-related diseases, providing valuable insights into how the pandemic has influenced public health behaviors and perceptions related to tobacco use. It is hoped that it can contribute to the development of more effective strategies for tobacco control and public health promotion in the post-pandemic era.

## 2 Methods

### 2.1 Sampling method and respondents

Due to the impact of the COVID-19 pandemic, the survey in 2022 was conducted using a convenience sampling method. The questionnaire surveys were carried out on site by trained interviewers. Multistage equal-proportion sampling was employed in 2024. First stratifying the target population by administrative districts, then randomly selecting neighborhoods proportional to their population size, and finally recruiting participants through household-based random sampling within each selected neighborhood. The respondents were permanent residents aged 15 years or older, who had resided in Rizhao City for at least 1 month prior to the survey.

### 2.2 Measures

The survey was conducted by trained investigators using the China Adult Tobacco Epidemic Questionnaire. After obtaining the informed consent of the respondents, information regarding their demographic characteristics, such as gender, age, education levels, occupation, and other basic information, was elicited from the respondents. The survey on the tobacco epidemic included information on smoking and passive smoking, as well as the respondents’ awareness of the hazards of tobacco. Current smokers were defined as individuals who smoked at the time of the survey, including both daily smokers and occasional smokers. The passive smoking rate refers to the proportion of people who are exposed to second-hand smoke for at least 1 day a week among non-smokers.

Four questions were included in the residents’ awareness of smoking-related diseases: (1) In your opinion, does smoking cause stroke (cerebral apoplexy, cerebral thrombosis, etc.)? (2) In your opinion, does smoking cause heart disease? (3) In your opinion, does smoking cause lung cancer? (4) In your opinion, does smoking cause penile erectile dysfunction? Three questions were included to assess non-smokers’ awareness of passive smoking-related diseases: (1) In your opinion, does passive smoking cause heart diseases in adults? (2) In your opinion, does passive smoking cause lung diseases in children? (3) In your opinion, does passive smoking cause lung cancer in adults? For each question, three response options were provided: “Yes,” “No,” and “Uncertain.” The awareness rate was calculated by dividing the number of respondents who chose “yes” by the total number of study participants.

### 2.3 Quality control

The investigation was conducted in strict compliance with the predefined research protocol. Before the survey was initiated, a team of experts conducted uniform training for all investigators and quality control personnel to ensure consistency and accuracy. During data sorting and analysis, a systematic sample review of the questionnaire was conducted in strict accordance with the established guidelines to maintain data integrity and reliability.

### 2.4 Statistical analysis

Data entry and analysis were performed using Excel 2010 and SPSS 27.0, respectively. Categorical data were described using percentages. The chi-square test was employed to compare statistical differences. All analyses were conducted using the two-tailed significance tests, and the hypothesis test’s significance level was set at *p* < 0.05.

## 3 Results

### 3.1 Current-smoking

As shown in Table 1 (Supplementary Figure 1), the current smoking rate in 2022 was 32.53% (*95%CI:* 29.34–35.72%), while it decreased to 25.84% in 2024 (*95%CI:* 23.22–28.46%), and the difference was statistically significant (*χ^2^* = 10.08, *p* = 0.002).

**Table 1:** Current smoking rates by sociodemographic groups, 2022 vs. 2024.

Categorical variables	2022		2024		χ^2^	*P*
	*n*	Current smoke rate (*95%CI*)	*n*	Current smoke rate (*95%CI*)		
Total	827	32.53(29.34–35.72)	1,045	25.84(23.22–28.46)	10.08	0.002
Gender						
Male	497	51.11(46.72–55.5)	622	37.14(33.53–40.75)	21.95	<0.01
Female	330	4.55(2.3–6.8)	423	9.22(6.41–12.03)	6.09	0.014
χ^2^		195.9		102.42		
*P*		<0.01		<0.01		
Age groups (years)						
15~	17	47.06(23.33–70.79)	64	37.50(25.47–49.53)	0.51	0.474
25~	59	25.42(14.31–36.53)	137	19.71(12.91–26.51)	0.80	0.371
35~	139	23.02(16.02–30.02)	138	24.64(17.20–32.08)	0.10	0.752
45~	228	28.95(23.06–34.84)	256	30.86(25.14–36.58)	0.21	0.647
55~	384	38.54(33.67–43.41)	450	23.56(19.76–27.36)	21.91	<0.01
χ^2^		16.38		11.93		
*P*		<0.01		0.018		
Education levels						
Junior high school or below	683	32.06(28.56–35.56)	616	22.73(19.45–26.01)	14.12	<0.01
High school degree	100	37.00(27.54–46.46)	235	33.19(27.27–39.11)	0.45	0.502
College degree or above	44	29.55(16.07–43.03)	194	26.80(20.51–33.09)	0.14	0.713
χ^2^		1.16		9.84		
*P*		0.561		<0.01		
Occupations						
Production personnel in agriculture, forestry, husbandry, and fisheries	439	36.45(31.95–40.95)	57	35.09(22.41–47.77)	0.04	0.841
Government/public institution staff	33	33.33(17.25–49.41)	140	28.57(21.08–36.06)	0.29	0.589
Employees of enterprises, businesses and service industries	98	32.65(23.37–41.93)	553	26.94(23.29–30.59)	1.35	0.245
Teachers and medical staff	16	18.75(0.38–37.88)	114	17.54(10.67–24.41)	0.01	0.906
Others (students, military, retired, etc.)	241	26.14(20.59–31.69)	181	22.65(16.65–28.65)	0.68	0.410
χ^2^		8.95		8.50		
*P*		0.062		0.075		

The prevalence of current smoking varied among residents with different demographic characteristics. The results of both surveys indicated that the current smoking rate among males was significantly higher than that among females (in 2020, *χ^2^* = 195.9, *p* < 0.01; in 2022, *χ^2^* = 102.42, *p* < 0.01). The current smoking rate among males showed a significant decline from 51.11 to 37.14% (*χ^2^* = 21.59, *p* < 0.01). Conversely, the current smoking rate among females exhibited an upward tendency (4.55% in 2022 vs. 9.22% in 2024, *χ^2^* = 6.09, *p* = 0.014).

From the perspective of age distribution, both surveys demonstrated that the current smoking rate was highest among residents aged 15–25 compared to other age groups: in 2022, the residents in the 15 ~ age group had the highest current smoking rate (47.06%), while the 35 ~ age group had the lowest (23.02%), and the difference was statistically significant (*χ^2^* = 16.38, *p* < 0.01); By 2024, while the 15 ~ age group still exhibited the highest current smoking rate at 37.50%, which was statistically higher than the 25 ~ age group (19.71%, *χ^2^* = 11.93, *p* = 0.018) A comparison of the data from the two surveys showed that the current smoking rate among residents aged 55 and above decreased significantly in 2024 (38.54% in 2022 vs. 23.56% in 2024, *χ^2^* = 21.91, *p* < 0.01).

Compared to 2022, the current smoking rates of residents at different education levels showed a downward trend in 2024. In 2022, the current smoking rate of the respondents with a junior high school education or below was 32.06%. However, in 2024, the current smoking rate of residents with a junior high school education or below was 22.73%, and the difference was statistically significant (*χ^2^* = 14.12, *p* < 0.01). The results of the two surveys both revealed that there were differences in the current smoking rates among residents of various occupational groups. In comparison with 2022, the current smoking rates in all occupational categories presented a downward trend in 2024.

### 3.2 Daily-smoking

As presented in Table 2 (Supplementary Figure 2), the daily smoking rate in 2022 was 20.44%, which decreased to 19.33% in 2024, with no statistically significant difference (*χ^2^* = 0.36, *p* = 0.551). Both surveys showed that the daily smoking rate among males was significantly higher than that among females. In 2022, the daily smoking rate of males was 32.39%, which was significantly higher than that of females (2.42%, *χ^2^* = 109.56, *p* < 0.01). In 2024, the daily smoking rate of males was 28.78%, while the rate of females was 5.44%, the difference was statistically significant (*χ^2^* = 87.96, *p* < 0.01). In terms of time, the daily smoking rate among males has decreased after the COVID-19 pandemic, while that among females showed an upward trend (*χ^2^* = 4.26, *p* = 0.039).

**Table 2:** Daily smoking rates by sociodemographic groups, 2022 vs. 2024.

Categorical variables	2022 (*N* = 827)		2024 (N = 1,045)		χ^2^	*P*
	Daily smoker	Rate (%,*95CI*)	Daily smoker	Rate (%,*95CI*)		
Total	169	20.44(17.80–23.07)	202	19.33(16.84–21.82)	0.36	0.551
Gender						
Male	161	32.39(28.22–36.60)	179	28.78(25.25–32.31)	1.71	0.191
Female	8	2.42(0.76–4.09)	23	5.44(3.26–7.62)	4.26	0.039
χ^2^	109.56		87.96			
*P*	<0.01		<0.01			
Age groups (years)						
15~	5	29.41(8.37–50.45)	14	21.88(11.84–31.92)	0.11	0.741
25~	8	13.56(5.02–22.10)	23	16.79(10.43–23.15)	0.32	0.570
35~	21	15.11(9.16–21.05)	34	24.64(17.19–32.09)	3.95	0.047
45~	45	19.74(14.58–24.90)	59	23.05(17.91–28.19)	0.78	0.376
55~	90	23.44(19.21–27.67)	72	16.00(12.66–19.34)	7.32	<0.01
χ^2^	7.18		8.80			
*P*	0.127		0.066			
Education levels						
Junior high school or below	143	20.94(17.97–23.91)	96	15.58(12.72–18.44)	6.18	0.013
High school degree	21	21.00(13.09–28.91)	63	26.81(21.31–32.31)	1.26	0.262
College degree or above	5	11.36(2.06–20.67)	43	22.16(16.30–28.02)	2.60	0.107
χ^2^	2.35		14.97			
*P*	0.308		<0.01			
Occupations						
Production personnel in agriculture, forestry, husbandry, and fisheries	108	24.60(20.61–28.60)	18	31.58(19.45–43.71)	1.30	0.255
Government/public institution staff	4	12.12(1.04–23.20)	28	20.00(13.37–26.63)	1.10	0.294
Employees of enterprises, businesses and service industries	19	19.39(11.66–27.11)	113	20.43(17.17–23.69)	0.06	0.812
Teacher and medical staff	2	12.50(0.00–28.56)	9	7.89(2.99–12.79)	0.02	0.890
Others (students, military, retired, etc.)	36	14.94(10.34–19.54)	34	18.78(13.02–24.54)	1.11	0.293
χ^2^	11.26		15.55			
*P*	0.024		<0.01			

Since the COVID-19 pandemic, the daily smoking rate among residents aged 55 and above has decreased significantly, dropping from 23.44% in 2022 to 16.00% in 2024 (*χ^2^* = 7.32, *p* < 0.01). The COVID-19 pandemic had varying impacts on the daily smoking rates among residents with different educational levels. It is noteworthy that the daily smoking rate of residents with a junior high school education or below has shown a significant decline, falling from 20.94% in 2022 to 15.58% in 2024 (*χ^2^* = 6.18, *p* = 0.013). The findings from both surveys consistently indicated that there were statistically significant differences in the daily smoking rates among residents of different occupations. Nevertheless, the COVID-19 pandemic exerted negligible influence on the daily smoking rates of residents within the same occupation.

### 3.3 Awareness of smoking-related diseases

The results showed that the order of residents’ awareness rates of smoking-related diseases, from high to low (lung cancer, heart diseases, stroke, and penile erectile dysfunction), was consistent in both surveys (Table 3 and Supplementary Figure 3). It is noteworthy that residents’ awareness rates of the aforementioned four smoking-related diseases have significantly decreased after the COVID-19 pandemic. Taking the residents’ awareness of whether smoking can cause lung cancer as an example, in 2022, 82.22% of the residents believed that smoking can cause lung cancer. However, in 2024, 77.70% of the residents held the same view, and the difference was statistically significant (*χ^2^* = 5.83, *p* = 0.016).

**Table 3:** Comparison of residents’ awareness of smoking-related diseases in 2022 and 2024.

Diseases	2022 (N = 827)		2024 (N = 1,045)		χ^2^	*p*
	*n*	Rate (%,*95CI*)	*n*	Rate (%,*95CI*)		
Stroke	559	67.59(64.38–70.81)	604	57.80(54.93–60.67)	18.82	<0.01
Heart diseases	563	68.08(64.88–71.27)	620	59.33(56.49–62.17)	15.19	<0.01
Lung cancer	680	82.22(79.80–84.65)	812	77.70(75.32–80.08)	5.83	0.016
Penile erectile dysfunction	387	46.80(43.36–50.23)	344	32.92(29.93–35.91)	37.35	<0.01

### 3.4 Passive smoking prevalence and awareness of passive smoking-related diseases

A statistically significant difference was observed between the passive smoking rates in 2022 and 2024 (32.08% in 2022 vs. 48.39% in 2024, *χ^2^* = 35.53, *p* < 0.01). Nevertheless, regarding residents’ awareness of passive smoking-related diseases, except that the awareness of “Lung diseases in children” remains basically stable, the awareness rates of all other smoking-related diseases showed a downward trend (Table 4 and Supplementary Figure 4).

**Table 4:** Comparison of Passive Smoking Rate and Residents’ Awareness of Passive Smoking-related Diseases among Non-smokers in 2022 and 2024.

Items	2022 (N = 558)		2024 (N = 775)		χ^2^	*p*
	*n*	Rate (%,*95CI*)	*n*	Rate (%*,95CI*)		
Passive smoking (≥1 days/week)	179	32.08(28.08–36.08)	375	48.39(44.88–51.90)	35.53	<0.01
Heart disease in adults	463	82.97(79.97–85.97)	479	61.81(58.39–65.23)	70.13	<0.01
Lung diseases in children	507	90.86(88.39–93.33)	723	93.29(91.35–95.23)	2.69	0.101
Lung cancer in adults	528	94.62(92.78–96.46)	709	91.48(89.29–93.67)	4.79	0.029

## 4 Discussion

According to the latest report on the Global Tobacco Epidemic, there are 1.3 billion tobacco users worldwide. More than 80% of those using tobacco products are in low- and middle-income countries, and up to half of the users are killed by tobacco (1). According to the Report on the Health Hazards of Smoking in China 2020, there were approximately 308 million smokers among Chinese aged 15 years and above (8). This data suggest that the tobacco epidemic is one of the greatest public health threats in China. According to the results of the National Adult Tobacco Epidemic Survey conducted in 2018, the prevalence of current smoking among adult residents in China was 26.6% (9). This study found that the current smoking rate among residents in Rizhao City in 2022 was higher than the national survey data, but it declined to a level below the national survey data by 2024. In 2022, factors such as increased stress, more time spent at home, and boredom during quarantine were commonly associated with the increase in tobacco use (10, 11). By 2024, the decrease in the current smoking rate may be closely linked to the effectiveness of tobacco control measures. Additionally, due to the economic downturn, residents’ consumption capacity was affected, leading to a reduction in tobacco consumption (12).

The survey results in both 2022 and 2024 showed that gender is a key factor influencing smoking behavior. This finding is consistent with extensive research. Influenced by sociocultural and biological factors, males tend to start smoking at an earlier age and have a consistently higher smoking prevalence across all age groups compared to females (13–16). However, our study showed a notable divergence: both the current smoking rate and daily smoking rate among female residents in Rizhao City in 2024 significantly surpassed 2022 levels. Potential explanations may include gender-specific marketing by tobacco industries, shifts in stress-coping mechanisms among women, or delayed policy impacts on female populations (17, 18).

Both the 2022 and 2024 surveys demonstrated age-specific disparities in current smoking rate and daily smoking rate. Residents aged 15 ~ had the highest smoking rates during both study periods. This might be associated with the exposure of youth to nicotine/tobacco-related content on social media platforms, which in turn elevates their risks of initiating tobacco product use (19). A tension between early emerging “bottom-up” systems that exhibit exaggerated reactivity to motivational stimuli and later maturing “top-down” cognitive control regions during adolescence may render the cognitive control processes more susceptible to modulation driven by incentives. Additionally, it can heighten the vulnerability to the alluring and rewarding properties inherent in alcohol and drugs (20–22).

By comparing the survey data of 2022 and 2024, it was found that the exposure of non-smokers to second-hand smoke significantly deteriorated in 2024. This result seemingly contradicts the decrease in the tobacco epidemic, and it may be explained through dual pathways: (1) The location shift effect: comprehensive smoke-free policies in public areas prompt smoking behavior to shift to residential areas and semi-enclosed transitional spaces (such as corridors) (23, 24). Moreover, during the pandemic, a substantial number of individuals were compelled to embrace remote work arrangements. Even in the post-pandemic period, such work patterns have endured, thereby resulting in a marked exacerbation of passive smoking exposure within both domestic and communal settings (25). (2) The population selection effect: after the COVID-19 pandemic, persistent smokers are more concentrated among socio-economically disadvantaged groups (26, 27). Factors such as higher household density (for example, living in shared apartments), working environments (such as enclosed workshops with poor ventilation), as well as a weaker awareness of the harms of tobacco, further exacerbate the risk of passive smoking for their family members and colleagues (6, 27, 28).

The public’s awareness of diseases caused by tobacco exposure and second-hand smoke exposure has decreased. Several factors may have contributed to this phenomenon: (1) Effect of public health resource depletion: during the COVID-19 pandemic, a large amount of publicity resources were allocated toward the prevention and control of respiratory diseases, resulting in a significant reduction in the exposure of tobacco harm education. (2) Weakened cognition among young people: the normalization of smoking behavior on social media may have weakened their awareness of tobacco harms. Meanwhile, respondents with limited knowledge about the health impacts on smoking and passive smoking were more likely to be exposed to second-hand smoke (29). Moreover, a study has revealed that although medical and dental students are fully aware of the hazards of cigarette smoking, they are reluctant to encourage habitual smokers to quit. This finding highlights the need for enhanced communication between smokers and non-smokers (30).

In conclusion, although the current-smoking rate in Rizhao has shown a declining trend, the decline in the daily smoking rate was not significant. Notably, the trend of tobacco use among the females has deteriorated, and residents’ awareness of the harms of tobacco and secondhand smoke exposure decreased. Therefore, to further promote tobacco control work, the following strategies are recommended: (1) carry out education on the hazards of tobacco for all residents: For example, leveraging social media and short-video platforms to disseminate tobacco control knowledge and the concept of a healthy lifestyle could effectively enhance public awareness and engagement. (2) Strengthen government-led policy enforcement: It is imperative to implement robust tobacco control policies, including raising tobacco taxes and ensuring strict enforcement of smoke-free laws in workplaces and public spaces (31). For instance, AI-driven monitoring systems could improve regulatory efficiency (31, 32). (3) Develop targeted interventions for females: we should focus on conducting in-depth investigations into the underlying reasons of smoking behavior changes among women and developing gender-specific prevention programs. (4) For adolescents, we should employ age-appropriate engagement strategies such as animated short films and educational games to deepen adolescents’ understanding of tobacco risks through culturally resonant formats.

By synergistically promoting these multifaceted measures, a tobacco-control atmosphere characterized by extensive societal participation and support can be cultivated. This will effectively contribute to reducing the prevalence of tobacco use and safeguarding public health.

### 4.1 Limitations

Owing to the strict lockdown policies imposed during the COVID-19 pandemic, the 2022 survey adopted a convenience sampling approach. Despite rigorous comparisons of gender and age distributions between the two surveys showing no statistically significant differences, considering the inherent limitations of the sampling method, readers are advised to interpret the findings with caution. In addition, this study was conducted exclusively among the residents of Rizhao. Given the region’s unique social, economic, and cultural environment as well as its population structure, the research findings may not be directly applicable to other areas of China. Additionally, it should be noted that based on the monitoring requirements of government departments, the participants in the 2022 and 2024 surveys were different. Future studies could focus on tracking the same population cohort to observe possible changes in smoking patterns before, during, and after the pandemic. Employing longitudinal approaches might provide more precise and actionable insights to better support targeted policy formulation and implementation.

## Data Availability

The raw data supporting the conclusions of this article will be made available by the authors, without undue reservation.
