# Supplementary material for: A comparative study on tobacco prevalence and secondhand smoke exposure before and after the lockdown in Rizhao, China: analysis of 2022 and 2024 data
Source: Front Public Health. 2025 Jun 26;13:1588781. doi: 10.3389/fpubh.2025.1588781 (PMC12241156; doi:10.3389/fpubh.2025.1588781)
Supplement: Supplementary file 1 [file Table_1.DOCX]

Supplement table 1 Multivariate analysis of factors influencing the current-smoking rate among residents

|  |  | 2022 | | | |  | 2024 | | | |
| --- | --- | --- | --- | --- | --- | --- | --- | --- | --- | --- |
| Variable | Reference Level | B | S.E | p-value | 95%CI |  | B | S.E | p-value | 95%CI |
| Gender | female | 2.421 | 0.202 | <0.01 | 7.571-16.720 |  | 1.74 | 0.19 | <0.01 | 3.929-8.263 |
| Age | 15~ | -0.156 | 0.071 | 0.028 | 0.745-0.983 |  | 0.078 | 0.065 | 0.227 | 0.953-1.228 |
| Education levels | Junior high school or below | 0.031 | 0.097 | 0.75 | 0.844-1.260 |  | -0.195 | 0.095 | 0.04 | 0.683-0.991 |
| Occupations | Others | 0.055 | 0.082 | 0.501 | 0.899-1.241 |  | 0.048 | 0.073 | 0.511 | 0.910-1.210 |
