# Supplementary material for: A comparative study on tobacco prevalence and secondhand smoke exposure before and after the lockdown in Rizhao, China: analysis of 2022 and 2024 data
Source: Front Public Health. 2025 Jun 26;13:1588781. doi: 10.3389/fpubh.2025.1588781 (PMC12241156; doi:10.3389/fpubh.2025.1588781)
Supplement: Supplementary file 2 [file Data_Sheet_1.PDF]

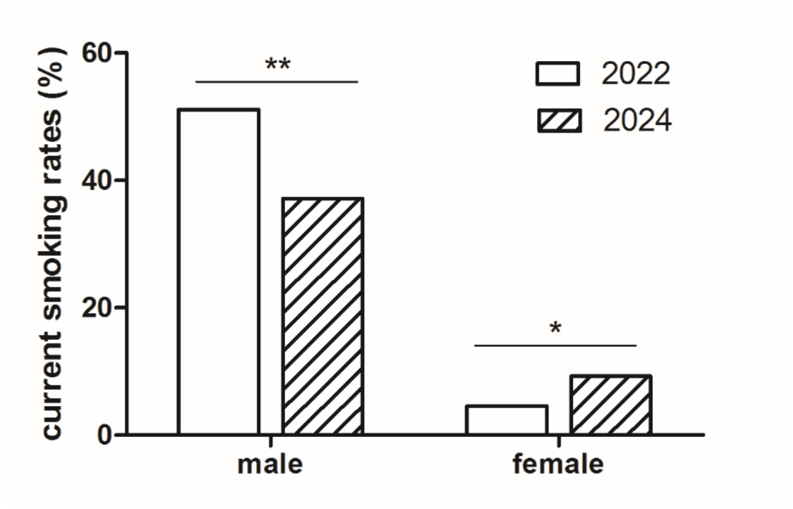

Supplementary Figure 1 Comparison of current smoking rates by gender in 2022 and 2024 (* P<0.05; ** P<0.01)

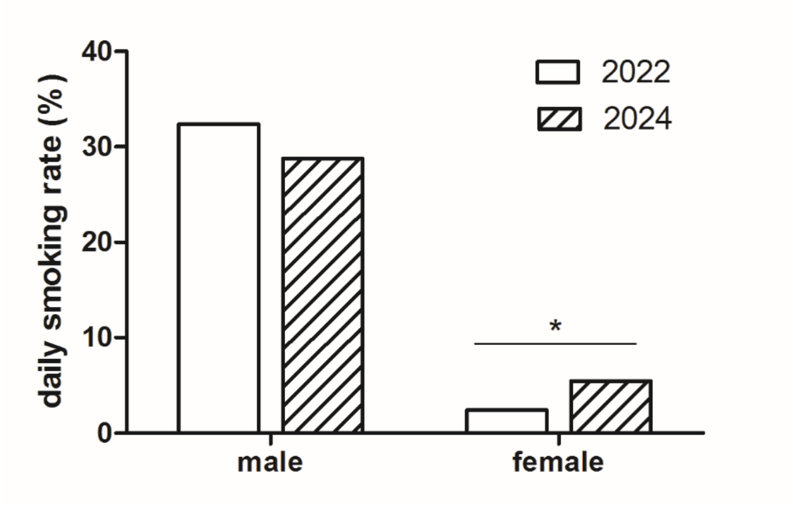

Supplementary Figure 2 Comparison of daily smoking rates by gender in 2022 and 2024 (* P<0.05)

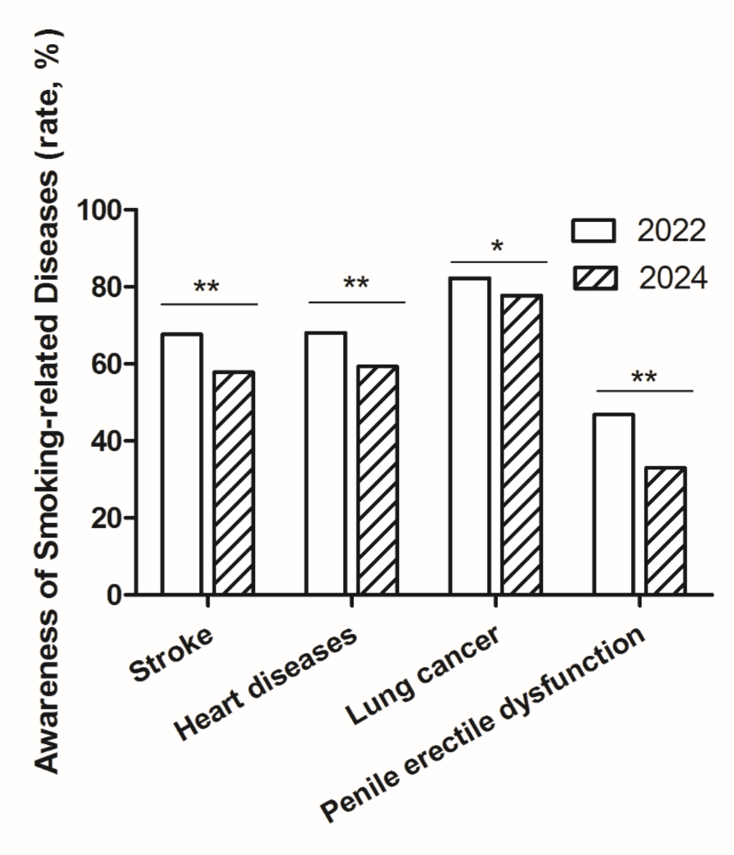

Supplementary Figure 3 Comparison of residents' awareness of smoking-related diseases in 2022 and 2024 (* $P<0.05$; ** $P<0.01$)

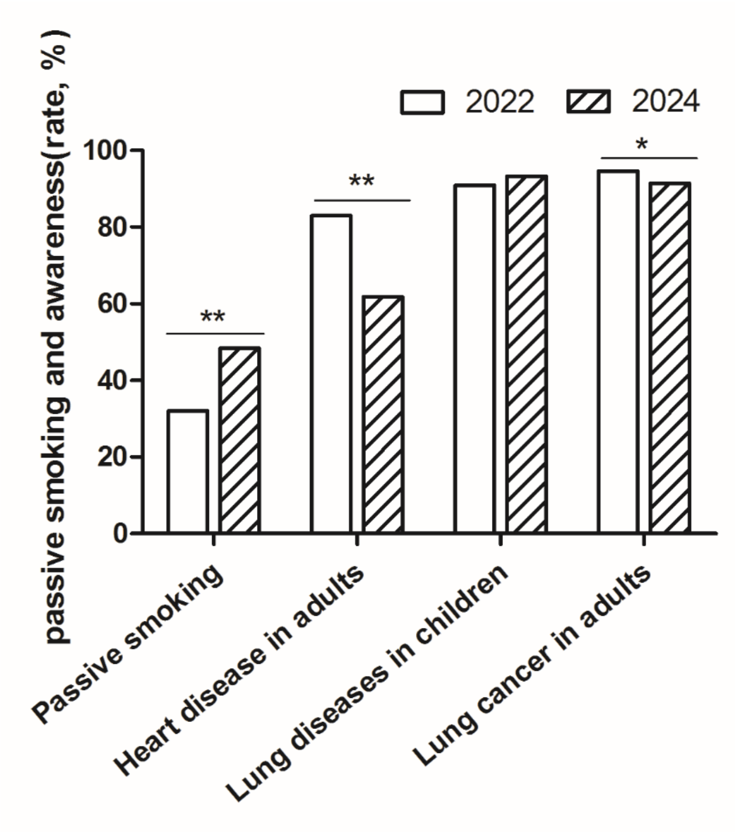

Supplementary Figure 4 Comparison of passive smoking rate and residents' awareness of passive smoking-related diseases among non-smokers in 2022 and 2024 (* P<0.05; ** P<0.01)
